# Cyanidin-3-*O*-Glucoside in Anthocyanin-Rich Fraction Combined with Atorvastatin Mitigates Kidney Injury via Attenuating Renal Lipotoxicity and Oxidative Stress and Preserving Mitochondrial Function in Obese Rats

**DOI:** 10.3390/ijms27219462

**Published:** 2026-10-23

**Authors:** Chorchat Lalichatsakul, Pattarasritida Phatthapong, Nattavadee Pengrattanachot, Prempree Sutthasupha, Pongrapee Laorodphun, Sarun Nakrat, Phumon Sookwong, Sugunya Mahatheeranont, Anusorn Lungkaphin

**Affiliations:** 1 Renal Transporter and Molecular Signaling Unit, Department of Physiology, Faculty of Medicine, Chiang Mai University, Chiang Mai 50200, Thailand; chorchat_lali@cmu.ac.th (C.L.); pattarasritida_ph@cmu.ac.th (P.P.); prempree.sut@cmu.ac.th (P.S.); pongrapee.l@cmu.ac.th (P.L.); 2 Department of Physiology, Faculty of Medicine, Kasetsart University, Bangkok 10900, Thailand; ntvd.peng@gmail.com; 3 Functional Foods for Health and Disease, Department of Physiology, Faculty of Medicine, Chiang Mai University, Chiang Mai 50200, Thailand; 4 Department of Chemistry, Faculty of Science, Chiang Mai University, Chiang Mai 50200, Thailand; sarun_n@cmu.ac.th (S.N.); phumon.s@cmu.ac.th (P.S.); sugunya.w@gmail.com (S.M.); 5 Center of Excellence for Innovation in Chemistry, Faculty of Science, Chiang Mai University, Chiang Mai 50200, Thailand; 6 Functional Food Research Center for Well-Being, Multidisciplinary Research Institute, Chiang Mai University, Chiang Mai 50200, Thailand

**Keywords:** obesity, kidney injury, cyanidin-3-*O*-glucoside, atorvastatin, lipotoxicity

## Abstract

Obesity-related kidney injury is associated with renal lipotoxicity, mitochondrial dysfunction, and oxidative stress. This study investigated the potential renoprotective effects of cyanidin-3-*O*-glucoside (C3G) in an anthocyanin-rich fraction from black glutinous rice (*Oryza sativa* L.) bran in combination with atorvastatin (ATO) in obese rats. Rats were fed a high-fat diet (HFD) for 16 weeks, followed by 8 weeks of treatment with C3G (100 or 200 mg/kg BW/day) combined with ATO (5 mg/kg BW/day) or ATO alone (10 mg/kg BW/day). HFD increased renal lipid accumulation and oxidative stress and altered mitochondrial-related proteins and renal structure. Both C3G doses combined with ATO 5 mg/kg BW/day improved selected metabolic and renal outcomes. Based on the overall responses, C3G 100 mg/kg BW/day + ATO 5 mg/kg BW/day was selected for subsequent molecular analyses. This combination was associated with reduced renal lipid accumulation and favorable changes in proteins related to lipid metabolism, mitochondrial homeostasis, and oxidative stress, including PPARα, Sirt-1, PGC-1α, MFN2, Parkin, NOX4, Keap1, and Nrf2. Renal MDA levels were also reduced, accompanied by preservation of renal architecture and attenuation of glomerular, tubular, and fibrotic injury. Across several measured outcomes, the renoprotective effects of C3G 100 mg/kg BW/day + ATO 5 mg/kg BW/day were broadly comparable to those of ATO 10 mg/kg BW/day. These findings support further investigation of C3G combined with ATO as a potential adjunctive strategy for obesity-associated kidney injury.

## 1. Introduction

Obesity, defined by increased body mass index (BMI ≥ 25 kg/m^2^) and visceral fat accumulation, is a major contributor to chronic kidney disease (CKD) and is strongly associated with proteinuria and reduced estimated glomerular filtration rate (eGFR) [1]. Obesity-related kidney injury arises from multiple interrelated mechanisms. Chronic high-fat diet (HFD) consumption induces adipose tissue hypertrophy and dysfunction, resulting in systemic metabolic disturbances and low-grade inflammation [2]. Excess circulating fatty acids (FAs) subsequently lead to ectopic fat deposition in non-adipose tissues, such as the kidney, resulting in lipotoxicity [3]. Proximal tubular cells rely heavily on fatty acid oxidation (FAO) to meet their high energy demands for active solute reabsorption and maintenance of renal function [3]. Obesity promotes the upregulation of cluster of differentiation 36 (CD36), a key receptor for long-chain FA uptake, which may increase renal lipid accumulation and disrupt mitochondrial oxidative metabolism [4]. These alterations can increase reactive oxygen species (ROS) generation, rendering tubular cells vulnerable to oxidative stress and injury [4].

Sirtuin-1 (Sirt-1), an NAD^+^-dependent deacetylase, has been shown to promote FAO and alleviate lipotoxic stress by regulating genes involved in energy metabolism. Peroxisome proliferator-activated receptor α (PPARα) is an important transcriptional regulator of FA uptake and oxidation in the kidney, and its dysregulation may impair FAO under metabolic stress [5]. In contrast, fatty acid synthase (FAS) is a key enzyme involved in de novo fatty acid synthesis, and increased lipogenic activity may further promote intracellular lipid accumulation [6]. An imbalance between FAO and lipid synthesis contributes to the accumulation of toxic lipid intermediates, including ceramides and diacylglycerols, thereby exacerbating renal lipotoxicity [7]. Sirt-1 also activates peroxisome proliferator-activated receptor γ coactivator-1α (PGC-1α), a central regulator of mitochondrial biogenesis. Impairment of the Sirt-1/PGC-1α axis may contribute to mitochondrial dysfunction and obesity-related kidney injury [8].

Maintenance of mitochondrial homeostasis also requires coordinated mitochondrial dynamics and removal of damaged mitochondria through mitophagy. Parkin, an E3 ubiquitin ligase, is a key mediator of mitophagy and contributes to the selective removal of damaged mitochondria, whereas mitofusin2 (MFN2) is an important regulator of mitochondrial fusion and mitochondrial network homeostasis [9,10]. Dysregulation of Parkin-mediated mitophagy and MFN2-dependent mitochondrial dynamics may impair mitochondrial quality control and promote ROS accumulation [9,10].

NADPH oxidase 4 (NOX4) is an important source of reactive oxygen species (ROS) in the kidney, and excessive NOX4-derived ROS production may contribute to oxidative damage [11]. The Kelch-like ECH-associated protein 1 (Keap1)/nuclear factor erythroid 2-related factor 2 (Nrf2) pathway is a major regulator of cellular antioxidant and cytoprotective responses. Impairment of this pathway may reduce cellular capacity to counteract oxidative stress and increase susceptibility to renal injury [12]. These interconnected metabolic and mitochondrial disturbances may ultimately compromise both glomerular and tubular structures, contributing to obesity-associated renal injury and functional impairment [13]. Histological alterations in the glomerular and tubular compartments may occur together with changes in molecular indicators of renal injury and epithelial integrity, including kidney injury molecule-1 (Kim-1) and E-cadherin, respectively [14,15]. Therefore, assessment of renal morphology together with these molecular markers can provide complementary evidence of obesity-associated kidney injury. These findings suggest that obesity-induced renal injury involves interconnected disturbances in lipid metabolism, mitochondrial homeostasis and oxidative stress. Thus, targeting these pathways may represent a promising strategy for mitigating obesity-associated renal injury.

Natural bioactive compounds have attracted considerable attention for their therapeutic potential, particularly in metabolic disorders. Cyanidin-3-*O*-glucoside (C3G), one of the major anthocyanins in the flavonoid family, has been shown to improve hyperglycemia and enhance insulin sensitivity and dyslipidemia in obese and diabetic rodent models [16,17]. C3G also alleviates renal fibrosis and inflammation while enhancing antioxidant defenses [18]. Our previous study demonstrated that an anthocyanin-rich fraction (100 and 200 mg/kg BW/day) significantly improved renal function in obese rats by enhancing insulin sensitivity, reducing oxidative stress and inhibiting apoptosis [19]. Overall, this evidence supports the potential use of C3G as a protective agent against obesity-related kidney injury.

Atorvastatin (ATO), a widely used lipid-lowering agent, inhibits 3-hydroxy-3-methylglutaryl-coenzyme A (HMG-CoA) reductase and reduces endogenous cholesterol synthesis [20]. In obese rat models, ATO (10 mg/kg BW/day) exerts renoprotective effects by attenuating inflammation via inhibition of nuclear factor-κB (NF-κB) signaling and reducing apoptosis [21]. Additionally, ATO has been shown to mitigate oxidative stress by modulating ferroptosis-related pathways and enhancing antioxidant defenses under high-glucose conditions [22]. However, long-term use of high-dose ATO has been associated with impaired mitochondrial function and reduced insulin sensitivity [23]. Therefore, strategies that enhance the efficacy of low-dose ATO while minimizing adverse effects are desirable.

In this study, we investigated the effects of C3G in an anthocyanin-rich fraction extracted from black glutinous rice (*Oryza sativa* L.) at 100 and 200 mg/kg BW/day in combination with ATO at 5 mg/kg BW/day in an HFD-induced obese rat model. Building on previous evidence of the beneficial effects of C3G alone, we explored C3G as a potential adjunct to ATO, an established lipid-lowering agent, to avoid undesired adverse effects. This combination was based on the multifactorial nature of obesity-induced kidney injury, which involves interconnected disturbances in lipid metabolism, oxidative stress, and mitochondrial homeostasis. While ATO primarily targets cholesterol biosynthesis through inhibition of HMG-CoA reductase, C3G has been reported to exert broader metabolic and antioxidant effects. Therefore, their combination was hypothesized to provide potentially complementary effects across these processes. C3G was combined with a lower ATO dose to explore whether C3G could provide additional metabolic and renal benefits in the context of lower-dose ATO treatment, with ATO 10 mg/kg BW/day included as the reference monotherapy treatment. The specific rationale for the selected C3G and ATO doses is described in the Section 4.

## 2. Results

### 2.1. Metabolic Parameters and Renal Function at 16 Weeks

**Figure 1:**
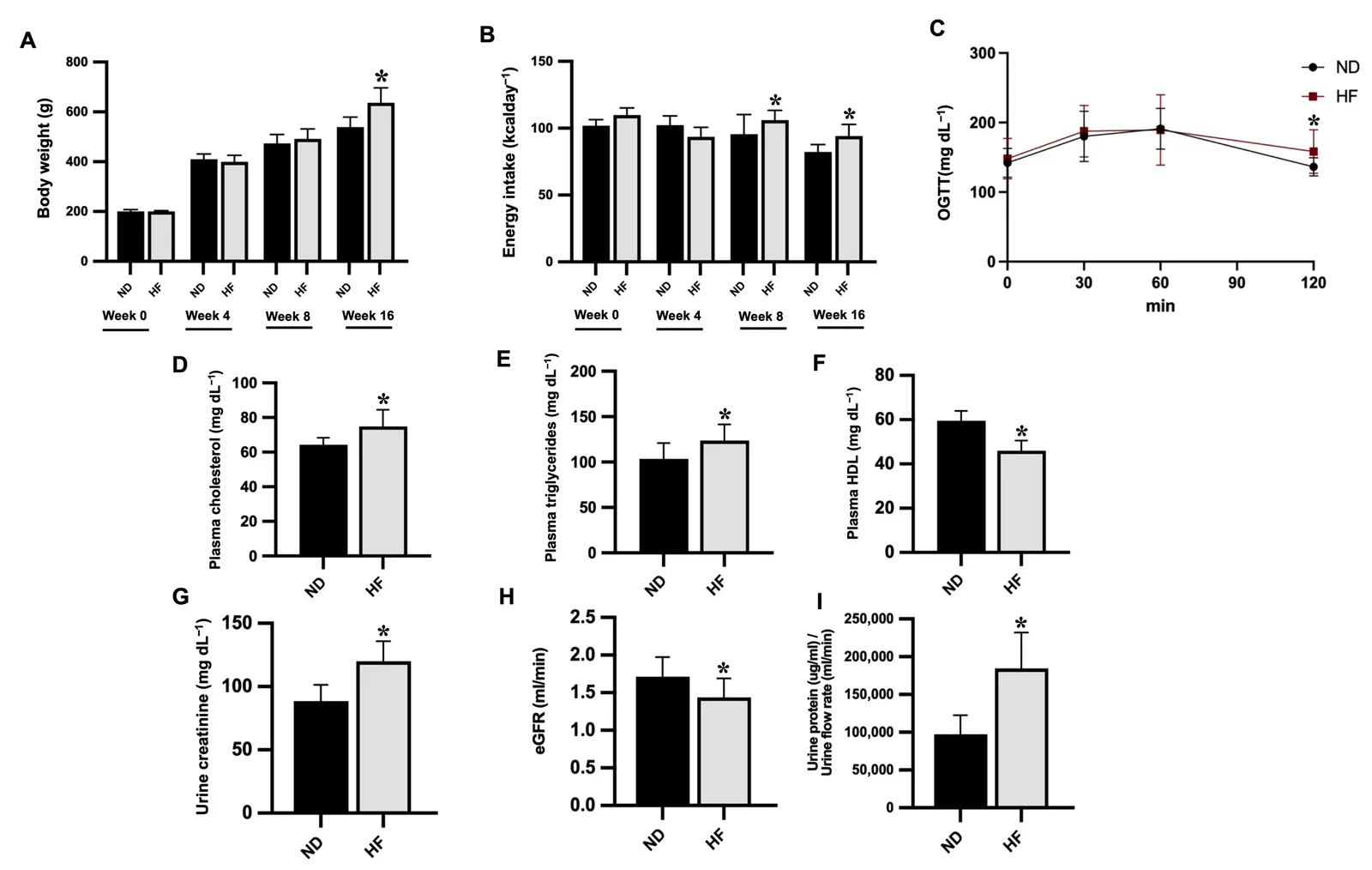
Metabolic parameters and renal function at 16 weeks. (**A**) body weight, (**B**) energy intake, (**C**) OGTT, (**D**) plasma cholesterol, (**E**) plasma triglycerides, (**F**) plasma HDL, (**G**) urine creatinine, (**H**) eGFR, (**I**) urine protein/urine flow rate. Data are presented as mean ± SD (ND, *n* = 12; HFD, *n* = 24). Data were normally distributed (Kolmogorov–Smirnov test, *p* > 0.05) and analyzed using an independent *t*-test. * *p* < 0.05 versus ND group. OGTT: oral glucose tolerance test; HDL: high-density lipoprotein; eGFR: estimated glomerular filtration rate; SD: standard deviation.

After 16 weeks of HFD feeding, an obese model was successfully established. HFD-fed rats exhibited significantly increased body weight, daily energy intake, dyslipidemia and impaired glucose tolerance, as indicated by elevated blood glucose at 120 min during OGTT, compared with normal diet (ND) rats (*p* < 0.05) (Figure 1A–F). In parallel, renal function had declined, reflected by an increase in serum creatinine and a reduction in eGFR compared with the ND rats (*p* < 0.05) (Figure 1G–I). These findings confirm that the HFD rats induced systemic metabolic disturbances and renal alterations.

### 2.2. The Impact of Combined C3G and ATO Treatment on Metabolic Parameters

**Figure 2:**
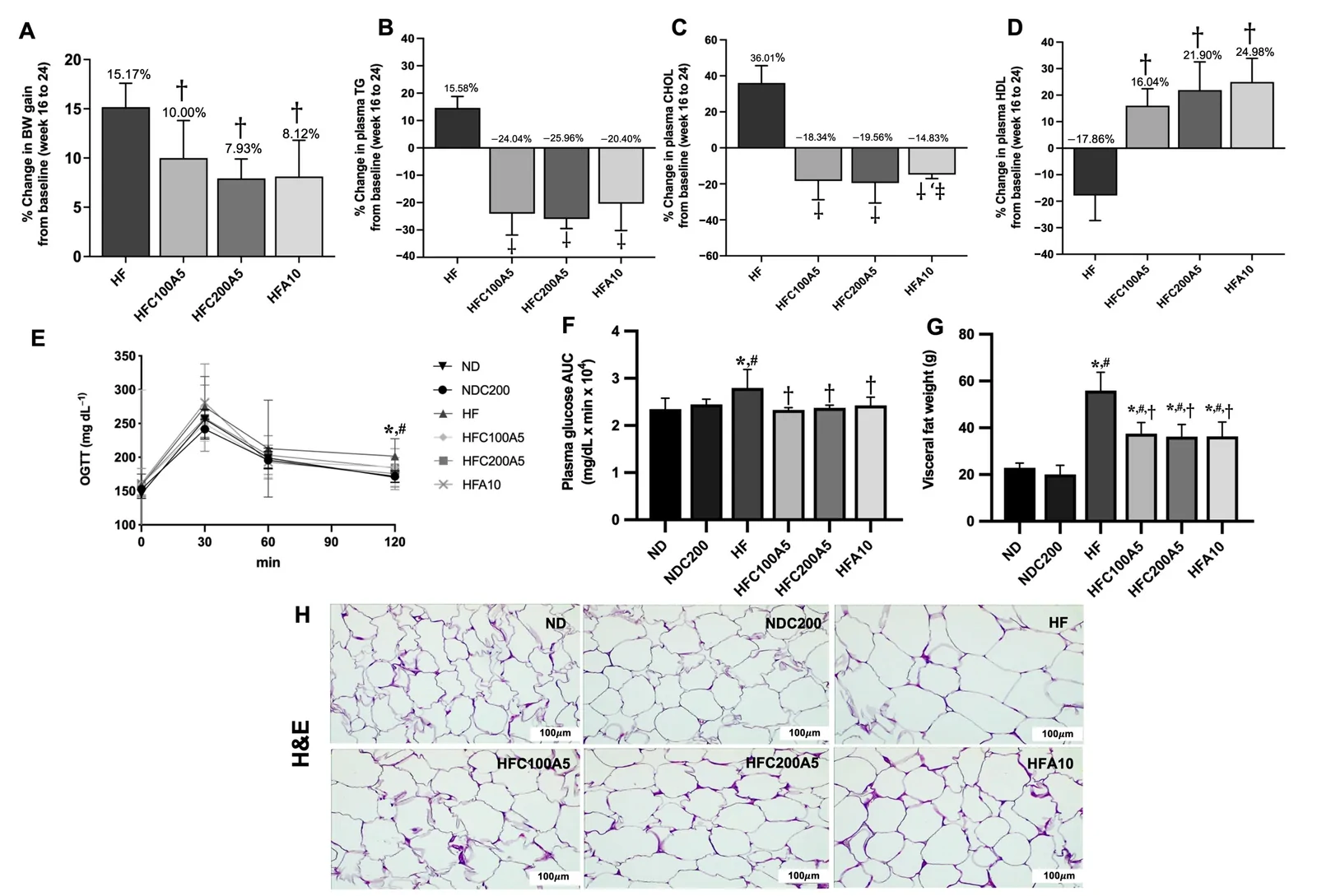
Effects of C3G and ATO treatment on the percentage change from weeks 16–24 in (**A**) BW gain, (**B**) plasma TG, (**C**) plasma CHOL and (**D**) plasma HDL. Results at week 24 for (**E**) OGTT, (**F**) plasma glucose AUC, (**G**) visceral fat weight and (**H**) adipocyte morphology. Data were normally distributed (Kolmogorov–Smirnov test, *p* > 0.05), are expressed as mean ± SD (*n* = 5–6 rats/group) and were analyzed using one-way ANOVA followed by Fisher’s least significant difference (LSD) post hoc test. * *p* < 0.05 versus ND group; # *p* < 0.05 versus NDC200 group; † *p* < 0.05 versus HF group; ‡ *p* < 0.05 versus HFC200A5 group. BW: body weight; TG: triglycerides; CHOL: cholesterol; HDL: high-density lipoprotein; OGTT: oral glucose tolerance test; AUC: area under the curve, H&E: hematoxylin and eosin. SD: standard deviation.

The percentage changes from baseline (week 16) to week 24 showed that treatment with C3G 100 mg/kg BW/day plus ATO 5 mg/kg BW/day (HFC100A5), C3G 200 mg/kg BW/day plus ATO 5 mg/kg BW/day (HFC200A5), and ATO 10 mg/kg BW/day (HFA10) significantly attenuated body weight gain and improved dyslipidemia compared with the HFD group (*p* < 0.05) (Figure 2A–D). HFC200A5 exhibited the lowest percentage of body weight gain (7.93%) among the treatment groups, followed by HFA10 (8.12%) and HFC100A5 (10.00%), although the differences were not statistically significant (Figure 2A). Moreover, both HFC100A5 (−24.04%) and HFC200A5 (−25.96%) exhibited a trend toward greater reductions in plasma triglyceride levels than HFA10 (−20.40%) (Figure 2B). Notably, HFC200A5 (−19.56%) showed a significantly greater reduction in plasma total cholesterol than HFA10 (−14.83%) (*p* < 0.05) (Figure 2C). In contrast, HFA10 (24.98%) exhibited a greater trend toward increasing plasma high-density lipoprotein (HDL) levels than HFC100A5 (16.04%) and HFC200A5 (21.90%) (Figure 2D).

At week 24, the HFD group exhibited significantly higher plasma glucose levels at 120 min during oral glucose tolerance tests (OGTTs) and a higher glucose area under the curve (AUC) than ND and ND treatment with C3G 200 mg/kg BW/day (NDC200) groups (*p* < 0.05) (Figure 2E,F). In contrast, HFC100A5, HFC200A5 and HFA10 exhibited a decreasing trend in plasma glucose levels during the OGTTs with a significant reduction in glucose AUC compared to the HFD group (*p* < 0.05) (Figure 2E,F). The HFD group also showed significantly increased visceral fat weight compared with the ND and NDC200 groups (*p* < 0.05), whereas all treatment groups significantly reduced visceral fat weight compared with the HFD group (*p* < 0.05) (Figure 2G). Hematoxylin and eosin (H&E) staining revealed smaller adipocytes with more compact morphology in all treatment groups, indicating attenuation of adipocyte hypertrophy (Figure 2H). Collectively, combined C3G and ATO (5 mg/kg BW/day) improved HFD-induced metabolic disturbances, including dyslipidemia, glucose intolerance and visceral adiposity. Moreover, HFC200A5 achieved a larger reduction in plasma cholesterol than HFA10, suggesting that C3G may enhance the lipid-lowering efficacy of ATO.

### 2.3. The Impact of Combined C3G and ATO Treatment on Renal Lipotoxicity

**Figure 3:**
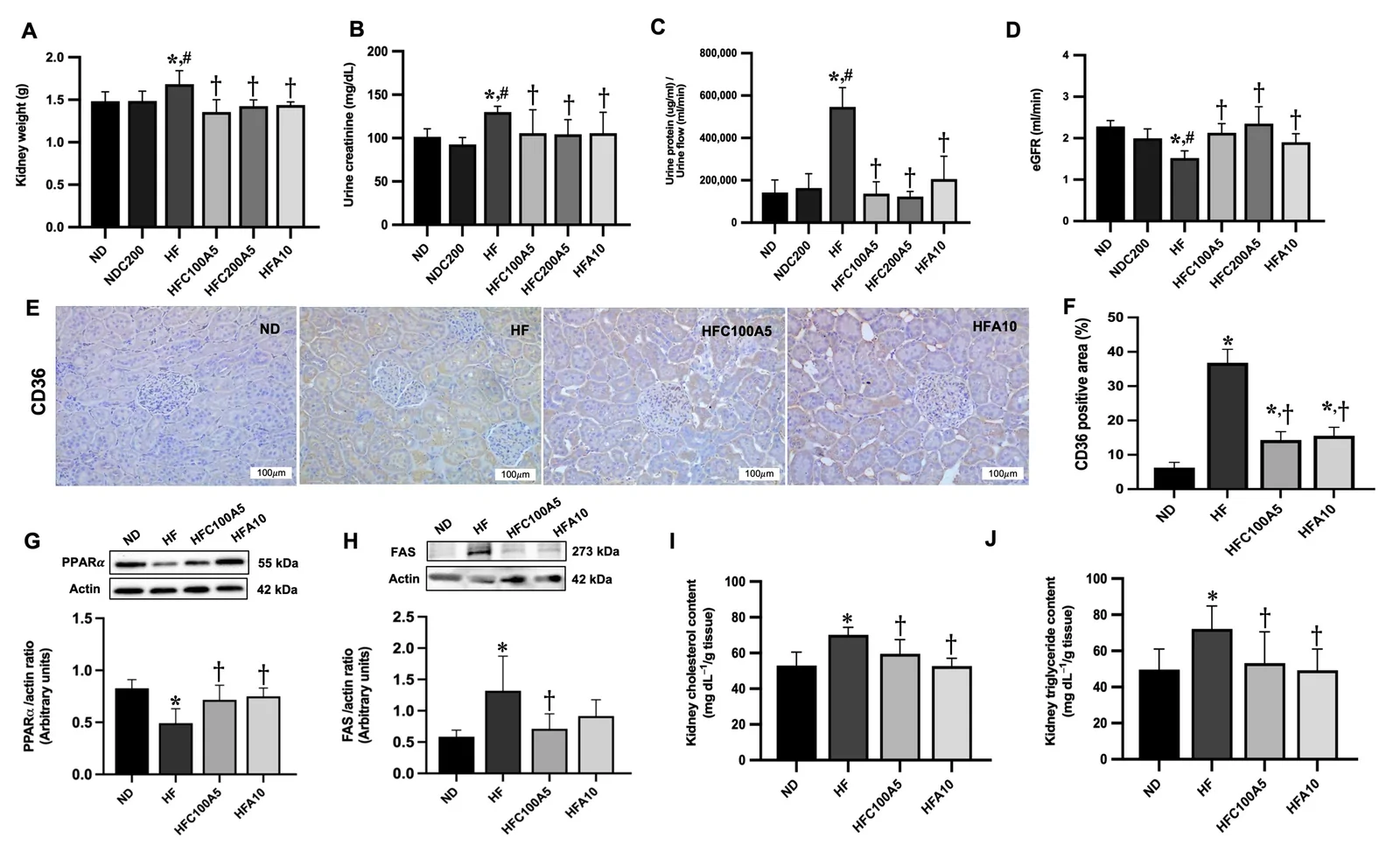
Effects of C3G and ATO treatment on renal lipotoxicity: (**A**) kidney weight, (**B**) urine creatinine, (**C**) urine protein, (**D**) estimated glomerular filtration rate (eGFR), (**E**) immunohistochemistry staining for cluster of differentiation 36 (CD36), (**F**) CD36 positive area, (**G**) peroxisome proliferator-activated receptor alpha (PPARα), (**H**) FAS, (**I**) kidney cholesterol content and (**J**) kidney triglyceride content. Data were normally distributed (Kolmogorov–Smirnov test, *p* > 0.05), are expressed as mean ± SD (*n* = 5–6 rats/group) and were analyzed using one-way ANOVA followed by Fisher’s least significant difference (LSD) post hoc test. * *p* < 0.05 versus ND group; # *p* < 0.05 versus NDC200 group; † *p* < 0.05 versus HF group. SD: standard deviation.

The HFD group exhibited clear signs of renal impairment, including increased kidney weight, urinary creatinine and protein excretion, along with a significant reduction in eGFR compared with the ND and NDC200 groups (*p* < 0.05). In contrast, all treatment groups showed significant improvement in renal function, as evidenced by reduced kidney weight, urinary creatinine and protein excretion, together with increased eGFR relative to the HFD group (*p* < 0.05) (Figure 3A–D). Based on the initial comparison of metabolic and renal outcomes among the treatment groups, the HFC100A5 group (C3G 100 mg/kg BW/day + ATO 5 mg/kg BW/day) was selected for subsequent molecular analyses. Although HFC200A5 showed a numerically greater reduction in body weight gain than HFC100A5, this difference was not statistically significant. HFC200A5 also produced a significantly greater reduction in plasma cholesterol; however, no significant differences were observed between HFC100A5 and HFC200A5 in the other major metabolic and renal functional outcomes considered for dose selection. Therefore, HFC100A5 was selected as the lower C3G dose because it produced broadly comparable overall metabolic and renal responses, while the higher C3G dose did not demonstrate a consistent additional benefit across the major outcomes evaluated. Subsequent molecular analyses were therefore performed specifically in the HFC100A5 group. The NDC200 showed no significant differences from ND in the evaluated renal function and metabolic parameters, indicating that C3G administration at 200 mg/kg BW/day did not produce detectable adverse effects under the conditions of the present study. This is consistent with previous reports in which C3G was administered to experimental animals at doses of 200 mg/kg BW/day without reported adverse effects [19]. Therefore, NDC200 was not included in the subsequent mechanistic analyses, which focused on molecular alterations associated with HFD-induced renal injury and their modulation by HFC100A5 and HFA10.

Immunohistochemical analysis demonstrated that renal CD36 expression was significantly elevated in the HFD-fed rats compared with the ND rats (*p* < 0.05) (Figure 3E). However, CD36 expression was significantly reduced in the HFC100A5 and HFA10 groups relative to the HFD group (*p* < 0.05). Consistent with impaired lipid metabolism, HFD-fed rats showed a marked decrease in PPARα expression compared with ND rats (*p* < 0.05), whereas both HFC100A5 and HFA10 treatments significantly restored PPARα levels (*p* < 0.05) (Figure 3F). Additionally, the HFD group exhibited significantly increased FAS expression and elevated renal triglyceride and cholesterol contents compared with the ND group (*p*< 0.05). Treatment with HFC100A5 reduced FAS expression, whereas HFA10 had no apparent effect on FAS expression. Nevertheless, both HFC100A5 and HFA10 significantly decreased renal triglyceride and cholesterol contents compared with the HFD group (*p* < 0.05) (Figure 3G–I). Overall, these findings indicate that both HFC100A5 and HFA10 were associated with attenuated renal lipotoxicity. The greater reduction in FAS expression observed with HFC100A5 compared with HFA10 may indicate a differential effect on this lipid synthesis-related protein.

### 2.4. Combined C3G and ATO Treatment on Renal Mitochondrial Biogenesis and Mitophagy

**Figure 4:**
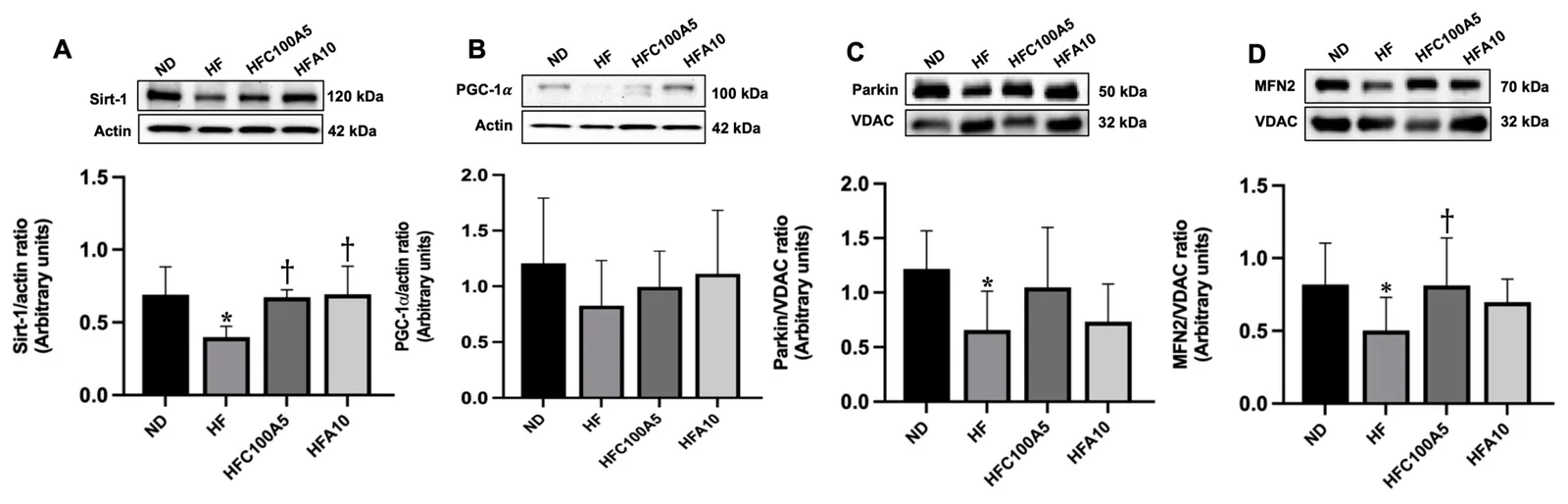
Effects of C3G and ATO treatment on mitochondrial function: (**A**) sirtunin-1 (Sirt-1), (**B**) peroxisome proliferator-activated receptor gamma coactivator-1alpha (PGC-1*α*), (**C**) Parkin and (**D**) mitofuscin2 (MFN2). Data were normally distributed (Kolmogorov–Smirnov test, *p* > 0.05), are expressed as mean ± SD (*n* = 5–6 rats/group) and were analyzed using one-way ANOVA followed by Fisher’s least significant difference (LSD) post hoc test. * *p* < 0.05 versus ND group; † *p* < 0.05 versus HF group. VDAC: voltage-dependent anion channel; SD: standard deviation.

The expression levels of Sirt-1 and PGC-1α were significantly lower in the HFD group compared to the ND group (*p* < 0.05) (Figure 4A,B). HFC100A5 and HFA10 treatments significantly increased Sirt-1 expression compared to the HFD group (*p* < 0.05). PGC-1α expression showed an upward trend in both treatment groups compared to the HFD group; however, these changes did not reach statistical significance (Figure 4A,B). Regarding mitochondrial dynamics and mitophagy-related protein, MFN2 and Parkin expression were significantly lower in the HFD group than in the ND group (*p* < 0.05) (Figure 4C,D). HFC100A5 treatment significantly increased MFN2 expression compared with the HFD group (*p* < 0.05), whereas HFA10 showed a non-significant increase. Both treatment groups showed an upward trend in Parkin expression compared with the HFD group, suggesting a potential improvement in mitophagy-related processes (Figure 4C,D). Overall, both HFC100A5 and HFA10 were associated with changes in proteins related to mitochondrial biogenesis, mitochondrial dynamics, and mitophagy-related processes, suggesting a potential partial improvement in mitochondrial homeostasis. Notably, HFC100A5 significantly increased MFN2 expression, suggesting a greater effect on mitochondrial fusion-related protein expression than HFA10.

### 2.5. Combined C3G and ATO Treatment on Renal Oxidative Stress

**Figure 5:**
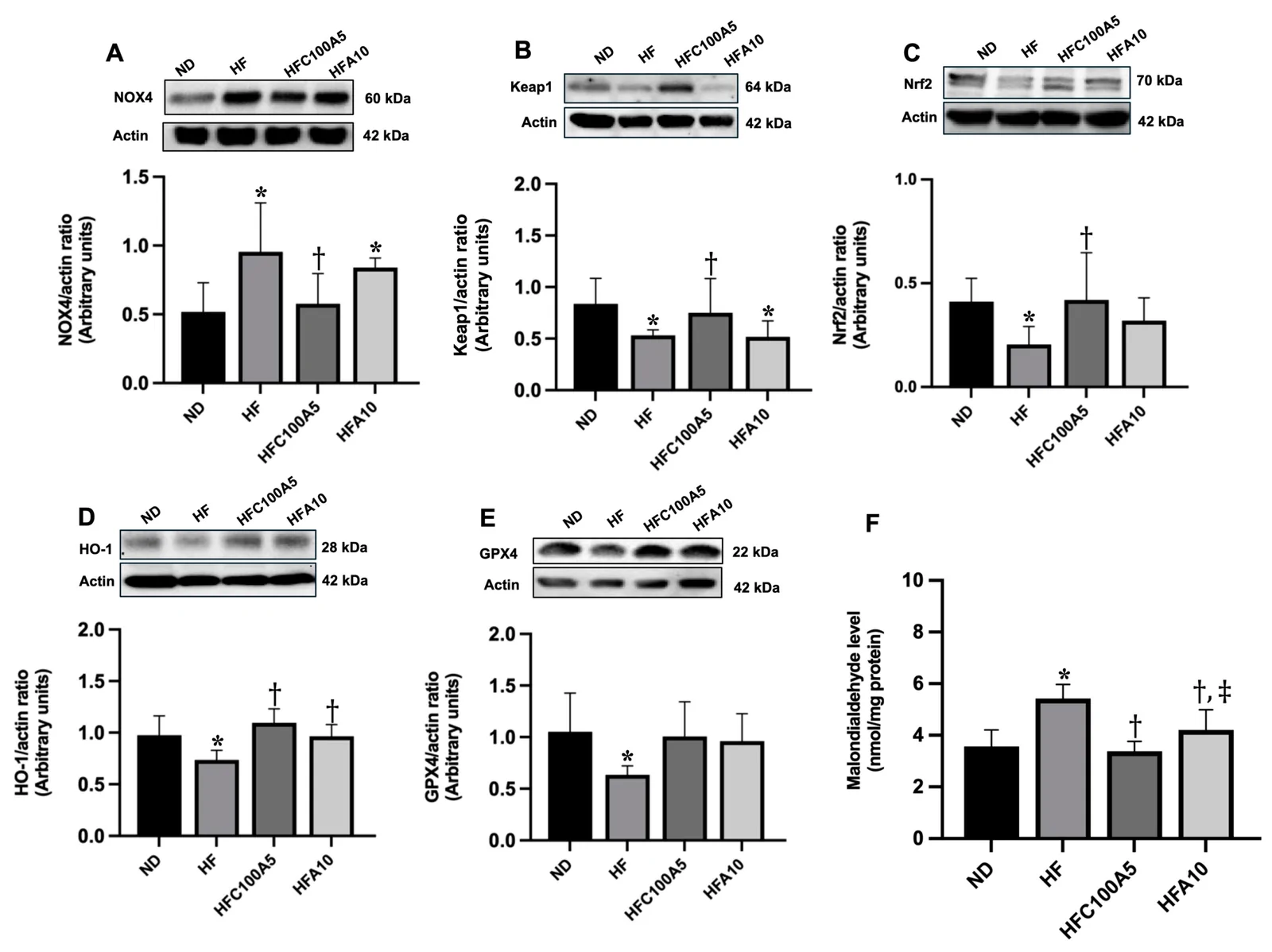
Effects of C3G and ATO treatment on oxidative stress: (**A**) nicotinamide adenine dinucleotide phosphate oxidase 4 (NOX4), (**B**) Kelch-like ECH-associated protein 1 (Keap1), (**C**) nuclear factor erythroid 2-related factor2 (Nrf2), (**D**) heme oxygenase-1 (HO-1), (**E**) glutathione peroxidase 4 (GPX4) and (**F**) malondialdehyde level. Data were normally distributed (Kolmogorov–Smirnov test, *p* > 0.05), are expressed as mean ± SD (*n* = 5–6 rats/group) and were analyzed using one-way ANOVA followed by Fisher’s least significant difference (LSD) post hoc test. * *p* < 0.05 versus ND group; † *p* < 0.05 versus HF group; ‡ *p* < 0.05 versus HFC100A5 group. SD: standard deviation.

The HFD group exhibited increased oxidative stress, as evidenced by significantly elevated NOX4 expression and reduced Keap1 and Nrf2 expression compared with the ND group (*p* < 0.05) (Figure 5A–C). These molecular alterations were accompanied by decreased heme oxygenase-1 (HO-1) and glutathione peroxidase 4 (GPX4) expression and increased malondialdehyde (MDA) levels relative to the ND group (*p* < 0.05) (Figure 5D–F). HFC100A5 treatment significantly reduced NOX4 expression and significantly increased Keap1 and Nrf2 expression compared with the HFD group (*p* < 0.05). In contrast, HFA10 did not significantly affect NOX4, Keap1, or Nrf2 expression (Figure 5A–C). Both HFC100A5 and HFA10 significantly increased HO-1 expression (*p* < 0.05) and showed an upward trend in GPX4 expression compared with the HFD group (Figure 5D,E). In addition, both treatment groups significantly reduced renal MDA levels compared with the HFD group (*p* < 0.05). Notably, HFC100A5 resulted in a significantly greater reduction in renal MDA levels than HFA10 (*p* < 0.05) (Figure 5F). Overall, these findings suggest that both HFC100A5 and HFA10 were associated with reduced renal oxidative stress, while HFC100A5 showed a greater effect on selected oxidative stress-related parameters, particularly NOX4 expression and renal MDA levels.

### 2.6. Combined C3G and ATO Treatment on Glomerular and Tubular Injury

**Figure 6:**
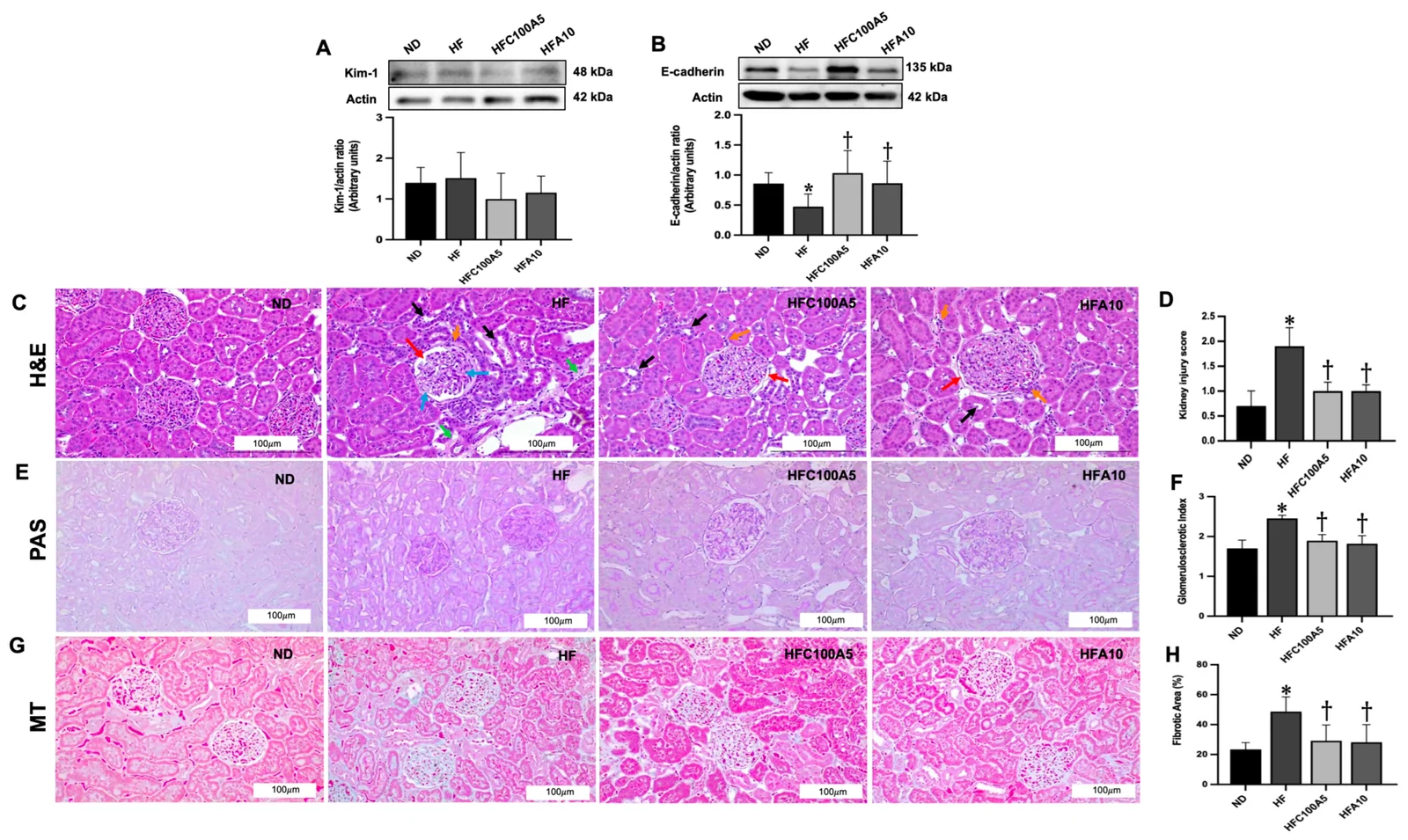
Effects of C3G and ATO treatment on kidney injury: (**A**) kidney injury-1 (Kim-1), (**B**) epithelial cadherin (E-cadherin), (**C**) renal histopathological alterations by hematoxylin and eosin (H&E) staining (red arrows: hypertrophy of Bowman’s capsule space; blue arrows: periglomerular fibrosis; green arrows: interstitial fibrosis; black arrows: tubular dilation; orange arrows: macrophage infiltration), (**D**) kidney injury score, (**E**) periodic acid-Schiff (PAS) staining, (**F**) glomerulosclerosis score, (**G**) Masson trichrome (MT) staining and (**H**) Masson’s %Positive area. Data were normally distributed (Kolmogorov–Smirnov test, *p* > 0.05), are expressed as mean ± SD (*n* = 5–6 rats/group), and were analyzed using one-way ANOVA followed by Fisher’s least significant difference (LSD) post hoc test. * *p* < 0.05 versus ND group; † *p* < 0.05 versus HF group.

The HFD group exhibited renal injury, as evidenced by significantly reduced E-cadherin, and tended to have increased Kim-1 expression compared with the ND group (*p* < 0.05) (Figure 6A,B). Both HFC100A5 and HFA10 significantly increased E-cadherin expression and showed a downward trend in Kim-1 expression compared with the HFD group, suggesting partial attenuation of tubular injury. Histopathological analysis supported these findings. H&E-stained kidney sections from the ND group displayed normal glomerular and tubular architecture, whereas the HFD group showed distorted glomeruli, dilated Bowman’s spaces, tubular dilation, peritubular and interstitial collagen deposition, and lymphocytic infiltration, corresponding to a significantly elevated kidney injury score (*p* < 0.05) (Figure 6C,D). Both treatment groups preserved renal structure and exhibited significantly lower kidney injury scores than the HFD group (*p* < 0.05) (Figure 6C,D). Periodic acid-Schiff (PAS) staining revealed thickened glomerular basement membranes and dense PAS-positive matrix accumulation in the HFD group, with a significantly elevated glomerulosclerosis index (*p* < 0.05) (Figure 6E,F). These pathology changes were markedly reduced by HFC100A5 and HFA10 (*p* < 0.05). Similarly, Masson’s trichrome staining showed extensive collagen deposition and fibrosis in the HFD group, both of which were significantly reduced by HFC100A5 and HFA10 (*p* < 0.05) (Figure 6H). These findings demonstrate that both HFC100A5 and HFA10 were associated with attenuation of HFD-induced renal structural injury and fibrosis. The comparable improvements observed across several renal injury and histopathological parameters suggest that HFC100A5 provided a degree of renal protection similar to that observed with HFA10 in the present study.

## 3. Discussion

The present study demonstrates that co-treatment with C3G and ATO (5 mg/kg BW/day) attenuated obesity-induced kidney injury. HFC100A5 was selected for subsequent molecular analyses as the lower C3G dose produced broadly comparable overall metabolic and renal responses to HFC200A5, whereas the higher C3G dose did not demonstrate a consistent additional benefit across the major outcomes evaluated. The subsequent molecular analyses were therefore performed specifically in the HFC100A5 group. At this dose, C3G co-treatment was associated with three major aspects of renal protection: (1) improved systemic lipid metabolism, as evidenced by a greater percentage reduction in plasma cholesterol during the treatment period compared with HFA10; (2) changes in protein associated with mitochondrial homeostasis, with HFC100A5 showing a greater increase in MFN2 expression than HFA10, suggesting a potential effect on mitochondrial dynamics; and (3) attenuation of renal oxidative stress, characterized by reduced NOX4 expression, increased Keap1 and Nrf2 expression, and a greater reduction in renal MDA levels compared with HFA10. The potential mechanisms underlying these renoprotective effects are discussed in the following sections.

Obesity-associated lipotoxicity arises from chronic excess energy intake, which initially activates peroxisome proliferator-activated receptor gamma (PPARγ) to promote adipose tissue expansion [24]. However, progressive adipose tissue dysfunction, characterized by hypoxia, insulin resistance, and inflammation, can impair lipid storage capacity and promotes FA spillover, leading to ectopic lipid accumulation in non-adipose tissues, including the kidney, and subsequent lipotoxic injury [25]. In the present study, the treatment groups showed anti-obesity effects, as evidenced by marked reductions in body weight gain, visceral fat weight, and adipocyte size. Previous studies have suggested that C3G and ATO may ameliorate obesity-related metabolic disturbances through AMP-activated protein kinase (AMPK)-dependent pathways, which can inhibit PPARγ activity and thereby suppress adipogenesis and lipogenesis [26,27]. In this study, co-treatment with C3G 200 mg/kg BW/day and ATO at 5 mg/kg BW/day showed a trend toward a greater reduction in the percentage change in body weight gain compared with HFA10. This observation is consistent with previous evidence that C3G may enhance energy expenditure through upregulation of uncoupling protein-1 (UCP-1) in brown adipose tissue. Increased UCP-1 promotes mitochondrial proton leak and dissipates energy as heat, thereby increasing energy expenditure and potentially limiting body-weight gain [28]. These findings suggest that C3G may be associated with additional effects on body-weight regulation.

Recent evidence indicates that obesity-associated elevations in extracellular FA promote CD36 trafficking to the plasma membrane, thereby enhancing the uptake of long-chain FA and oxidized low-density lipoprotein and potentially exceeding tubular metabolic capacity [29]. Consistent with this concept, the HFD group in the present study exhibited significantly increased renal CD36 expression. Both HFC100A5 and HFA10 significantly reduced CD36 expression, suggesting that these treatments may attenuate excessive renal lipid uptake associated with obesity. In diabetic kidney disease, CD36 overexpression has also been associated with impaired FAO, whereby excessive FA influx may exceed mitochondrial oxidative capacity and contribute to increased ROS production [30]. Oxidative stress may further impair PPARα signaling and FAO, thereby contributing to renal lipid dysregulation and injury [31]. Consistent with these findings, HFD rats exhibited significantly reduced PPARα expression compared with the ND group, whereas both HFC100A5 and HFA10 significantly restored PPARα expression. A previous study demonstrated that ATO activates the PPARα promoter and increases PPARα mRNA expression in HepG2 cells [32]. Similarly, C3G has been reported to act as a PPAR agonist with relatively high affinity for PPARα, thereby increasing the expression of PPARα target genes, including Acox1 and Ucp2, in insulin-resistant mice [33]. Together, these findings support the involvement of PPARα-associated FA metabolism in the effects of C3G and ATO observed in the present study.

A previous study in a diabetic model reported that C3G suppresses lipogenic markers, including FAS [34]. In contrast, ATO at 10 mg/kg BW/day did not significantly alter renal FAS expression in the present study. This may be related to the primary pharmacological action of ATO, which inhibits HMG-CoA reductase and thereby primarily reduces cholesterol biosynthesis rather than directly suppresses de novo lipogenesis. FAS expression is predominantly regulated by pathways involving sterol regulatory element-binding protein-1c (SREBP-1c) and insulin signaling [35]. Differences in treatment duration and tissue-specific responses may also contribute to the observed finding. Nevertheless, HFA10 significantly reduced renal lipid accumulation despite the absence of significant change in FAS expression, suggesting that its effects on renal lipid accumulation may involve mechanisms other than direct suppression of FAS. In contrast, HFC100A5 significantly reduced renal FAS expression, indicating an association with reduced expression of this lipogenic protein. Overall, the increased PPARα expression observed in both HFC100A5 and HFA10, together with reduced renal lipid accumulation, suggests improved regulation of renal lipid metabolism in both treatment groups.

Notably, HFC200A5 produced a greater percentage reduction in plasma cholesterol during the treatment period than HFA10. This finding is consistent with a recent study reporting that C3G suppresses cholesterol synthesis through modulation of the ROS/SREBP-2/HMG-CoA reductase signaling pathway, thereby reducing intracellular cholesterol accumulation under oxidative stress conditions [36]. Thus, the greater reduction in plasma cholesterol observed with HFC200A5 may be associated with the potential contribution of C3G to cholesterol metabolism when combined with ATO. Although HFC200A5 produced a greater reduction in plasma cholesterol than HFC100A5, this difference was not statistically significant. This finding suggests that the higher C3G dose may provide an additional effect on cholesterol metabolism.

Disruption of mitochondrial homeostasis is a key contributor to obesity-induced kidney injury. Sirt-1 plays a central role in the maintenance of mitochondrial homeostasis [37]. One of the downstream targets of Sirt-1 is PGC-1α, a master regulator of mitochondrial biogenesis, and deacetylation and activation of PGC-1α stimulate transcription of nuclear-encoded mitochondrial genes involved in oxidative phosphorylation, electron transport, and antioxidant defense [38]. Disruption of this pathway, as seen in conditions of obesity, is largely driven by metabolic overload. The excessive nutrient flux elevates the production of NADH and reduces NAD^+^ availability, lowering the NAD^+^/NADH ratio required for Sirt-1 activation [39]. These events diminish Sirt-1 activity, leading to impaired deacetylation and reduction in PGC-1α and ultimately reducing mitochondrial biogenesis [40]. In this study, we demonstrated that HFC100A5 and HFA10 significantly increased the level of expression of Sirt-1 and showed an upward trend in PGC-1α levels. A previous study reported that C3G activates Sirt-1 in an adult human hepatoma cell line (HuH7), leading to upregulation of PGC-1α and downstream mitochondrial biogenesis genes, including nuclear respiratory factor 1 (NRF1) and mitochondrial transcription factor A (TFAM) [41]. Regarding ATO, a recent study reported that ATO mitigates amyloid-β_1_-_42_-induced neurotoxicity by increasing expression of Sirt-1, thereby reducing oxidative stress and promoting autophagosome formation [42].

As mentioned earlier, the reduction in PGC-1α under HFD conditions impairs mitochondrial biogenesis and consequently disrupts the regulation of mitochondrial dynamics, including both fission and fusion processes. MFN2 is a GTPase located on the outer mitochondrial membrane that mediates mitochondrial fusion, a process essential for mixing mitochondrial contents, diluting damaged components, and maintaining mitochondrial morphology and inter-organelle communication [43]. Reduced expression of MFN2, as observed in obesity-related kidney injury, leads to mitochondrial fragmentation, impaired ATP synthesis, and enhanced susceptibility to apoptosis [44]. Importantly, beyond the role in mitochondrial fusion, MFN2 also acts as a receptor for Parkin during mitophagy [45]. Under conditions of mitochondrial depolarization, the kinase PINK1 phosphorylates MFN2 at threonine-111 and serine-442, enabling the recruitment of Parkin to the outer mitochondrial membrane [45]. This interaction initiates the ubiquitination and autophagic clearance of damaged mitochondria, a key quality control process that maintains the health of the mitochondrial population [46]. To our knowledge, this study provides the first in vivo evidence in an obese rat model that treatment with C3G in combination with ATO was associated with a significant increase in MFN2 expression and a trend toward increased Parkin expression. These findings suggest that the combination treatment may be associated with changes in proteins related to mitochondrial dynamics and mitophagy during obesity-induced kidney injury.

Several studies have demonstrated the beneficial effects of C3G on mitochondrial function. Obese mice receiving C3G (1 mg/mL in drinking water) exhibited increased thermogenesis in brown adipose tissue through upregulation of UCP-1, FA oxidation genes, and mitochondrial biogenesis markers [47]. In a fatty liver model, C3G treatment enhanced mitophagy as a consequence of increased expression of PINK, leading to a reduction in oxidative stress and apoptosis and ultimate reduction in the progression of steatosis [48]. Together, our findings suggest that C3G (100 mg/kg BW/day) plus ATO (5 mg/kg BW/day) may have complementary effects on proteins related to mitochondrial dynamics and mitophagy, potentially contributing to the maintenance of mitochondrial homeostasis during obesity-induced kidney injury.

In the kidney, excessive lipid accumulation and mitochondrial impairment result in electron leakage from the respiratory chain, generating ROS [49]. Among the major enzymatic sources of ROS, NOX4 is abundantly expressed in renal tubular cells, where it contributes to oxidative stress in conditions of metabolic overload [50]. Persistent activation of NOX4 promotes lipid peroxidation and cellular apoptosis, thereby amplifying renal injury [51].

Another key finding of this study was that HFC100A5 showed greater effects on selected indicators of renal oxidative stress than HFA10, as evidenced by reduced NOX4 expression, increased Keap1 and Nrf2 expression, and a greater reduction in renal MDA levels. C3G belongs to the anthocyanin class of flavonoids, characterized by a flavylium cation backbone with multiple hydroxyl groups that confer strong redox potential [52]. The catechol moiety on the B-ring (3′,4′-dihydroxyl groups) is particularly important for direct radical scavenging, donating hydrogen atoms or electrons to neutralize ROS such as superoxide anion (O_2_•^−^) and hydroxyl radicals (•OH) [53]. Thus, C3G provides dual antioxidant protection by directly neutralizing ROS through redox-active hydroxyl groups and indirectly strengthening endogenous defenses via Nrf2 activation, supporting the ability of C3G to restore renal redox homeostasis.

Central to this antioxidant protection is the Keap1/Nrf2 signaling pathway, a key regulator of cellular redox homeostasis. Upon oxidative challenge, Keap1 undergoes conformational modification, releasing Nrf2 to translocate into the nucleus, where it binds to antioxidant response elements (AREs) and induces transcription of detoxifying and antioxidant genes [54]. In this study, the combination treatment showed a significant increase in the expression of Keap1 along with activation of the endogenous antioxidant system. This coordinated upregulation likely reflects stabilization of the Keap1/Nrf2 complex to prevent excessive ROS signaling while maintaining redox homeostasis.

The suppression of NOX4 in the HFA10 group was not significant in this study. The proposed mechanism is that statins can reduce the biosynthesis of Coenzyme Q10 (CoQ10), an essential electron carrier and antioxidant within the mitochondrial respiratory chain [55]; reduced CoQ10 availability may impair electron transport efficiency, further contributing to ROS production [56]. Excessive ROS may further stimulate NOX4 expression, potentially creating a feed-forward loop. Nevertheless, HFA10 attenuated oxidative damage by enhancing endogenous antioxidant defenses. The upregulation of HO-1 and GPX4 likely increased the capacity to detoxify ROS and lipid hydroperoxides, thereby reducing lipid peroxidation, as reflected by lower MDA levels. These findings suggest that the antioxidant effects of ATO may be associated more strongly with changes in cellular antioxidant defenses than changes in NOX4 expression. In contrast, HFC100A5 may influence multiple oxidative stress-related processes, including NOX4-associated ROS generation and antioxidant defense, which may contribute to the attenuation of obesity-induced renal oxidative stress.

Obesity-induced renal injury involves progressive disruption of both glomerular and tubular integrity, ultimately leading to impaired renal function and fibrotic remodeling [57]. In the present study, HFD feeding reproduced key features of obesity-associated nephropathy, supporting the validity of this model for evaluating therapeutic interventions. Renal injury in obesity is largely driven by interconnected processes, including lipid accumulation, oxidative stress, mitochondrial dysfunction, and chronic cellular stress, which collectively promote tubular epithelial damage and extracellular matrix deposition.

Importantly, HFC100A5 was associated with preservation of renal structural integrity and improvement in selected indicators of renal function, with renal protective effects broadly comparable to those observed with HFA10 across several measured outcomes. These findings were accompanied by improvements in metabolic parameters, reductions in renal lipid accumulation, and changes in proteins associated with mitochondrial homeostasis and oxidative stress. Together, these alterations may contribute to the observed attenuation of renal injury and fibrosis, although causal relationships between these molecular changes and the renal protective effects cannot be established from the present study. The comparable renal protective effects observed with HFC100A5 and HFA10 support further investigation of C3G in combination with ATO 5 mg/kg BW/day as a potential adjunctive approach for obesity-associated kidney injury.

A limitation of the present study is the absence of HFD groups receiving C3G or ATO at 5 mg/kg BW/day alone, which prevents determination of the independent contribution of each treatment component and formal assessment of additive or synergistic interactions. In addition, the molecular analyses were based on protein expression without functional or loss-of-function approaches; therefore, the findings support possible associations with lipid metabolism, mitochondrial homeostasis, and antioxidant defense but do not establish pathway activation or causality. Further in vivo studies incorporating appropriate monotherapy groups and functional mechanistic approaches are warranted to clarify the independent and combined effects of C3G and ATO.

## 4. Materials and Methods

### 4.1. Extraction and Characterization of C3G-Enriched Fraction

The C3G-rich extract was provided by the Department of Chemistry, Faculty of Science, Chiang Mai University. Black glutinous rice bran (*Oryza sativa* L. cv. Luem Pua; IRGC 48315), harvested in Phayao, Thailand, was dried at 40 °C to a moisture content below 14% and milled to obtain rice bran. The bran was sieved and extracted with 80% ethanol containing 0.1% formic acid at 30 °C for 3 h. The resulting extract was filtered and concentrated under reduced pressure, followed by defatting with hexane to remove lipophilic components. The solvent was subsequently evaporated to yield a crude extract (40 g from 500 g of bran). The crude extract was further purified using Amberlite^®^ XAD7HP resin (Sigma-Aldrich, St. Louis, MO, USA), and anthocyanins were eluted with ethanol to obtain a C3G-enriched fraction containing approximately 785 mg of C3G per gram of extract. This fraction was subsequently subjected to chromatographic purification using silica gel column chromatography and reverse-phase high-performance liquid chromatography (RP-HPLC) on a C18 column. Fractions were monitored by thin-layer chromatography and ultraviolet–visible (UV-Vis) spectroscopy, and anthocyanin-rich fractions were pooled and further purified. The major compound was detected at approximately 520 nm and collected for further analysis. Structural identification was confirmed by mass spectrometry and nuclear magnetic resonance (NMR) spectroscopy, verifying the compound as C3G. All extracts and purified fractions were stored at −20 °C in the dark until use.

### 4.2. Experimental Protocol

**Figure 7:**
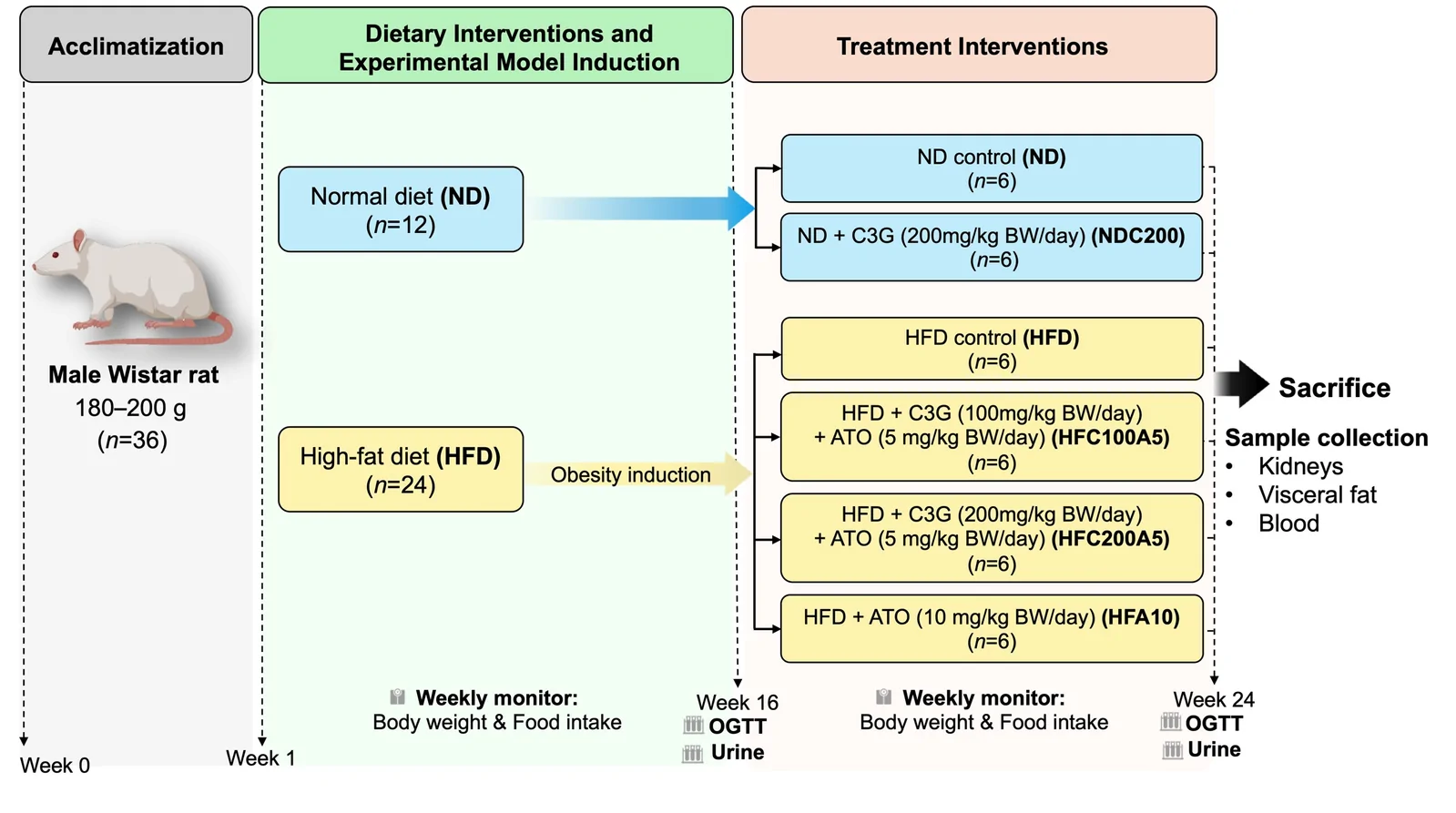
Schematic overview of experimental protocol. Blue boxes indicate normal-diet (ND) groups, yellow boxes indicate high-fat-diet (HFD) groups, the green background indicates the dietary intervention and experimental model induction phase, and the peach background indicates the treatment intervention phase. C3G: cyanidin-3-*O*-glucoside; ATO: atorvastatin; OGTT: oral glucose tolerance test. Created in BioRender. CMU, P. (2026) https://BioRender.com/m9s8ylk.

Male Wistar rats (*n* = 36, 180–200 g), approximately 8 weeks old at the beginning of the study, were obtained from Nomura Siam International (Bangkok, Thailand). Experimental procedures were approved by the Institutional Animal Care and Use Committee, Faculty of Medicine, Chiang Mai University, and followed National Institutes of Health guidelines for the ethical treatment of laboratory animals (Approval number 27/2566; approved from 21 August 2023 to 20 August 2024). Rats were housed under controlled conditions (25 ± 0.5 °C, 12 h light/dark cycle) with free access to standard diet and water and allowed to acclimatize for one week. Following acclimatization, the rats were randomly assigned into two groups. A first group continued receiving the standard commercial rodent diet (rodent fed food no. 082, C.P. Company, Bangkok, Thailand) (normal diet (ND), *n* = 12; 19.77% energy from fat), whereas a second group received a modified homemade HFD (HFD, *n* = 24; 59.28% energy from fat) [58]. Both dietary interventions were maintained throughout the 24-week experimental period. After 16 weeks of dietary intervention, the ND group was further divided into two subgroups: ND control and ND + C3G (200 mg/kg BW/day) groups (*n* = 6 per group). The HFD group was subdivided into four treatment groups (*n* = 6 per group): HFD control, HFD + C3G 100 mg/kg BW/day + ATO 5 mg/kg BW/day (HFC100A5), HFD + C3G 200 mg/kg BW/day + ATO 5 mg/kg BW/day (HFC200A5), and HFD + ATO 10 mg/kg BW/day (HFA10). C3G and/or ATO treatments were initiated at week 16 and administered by oral gavage once daily for 8 weeks, from weeks 16 to 24. All treatments were dissolved in distilled water. The C3G-enriched extract contained approximately 785 mg C3G/g extract (78.5% C3G by weight). Therefore, the administered doses of 100 and 200 mg/kg BW/day of C3G-enriched extract corresponded to approximately 78.5 and 157 mg C3G/kg BW/day, respectively. The C3G doses used in this study were selected based on previous studies demonstrating lipid-lowering and pleiotropic effects, including antioxidant and nephroprotective effects [19]. The ATO dose of 10 mg/kg BW/day was selected based on previous studies reporting lipid-lowering and renoprotective effects in an obese rat model, within established safety ranges [21,59]. In addition, a previous study in HFD-induced hypercholesterolemic rats demonstrated that ATO at 5 mg/kg BW/day significantly reduced plasma cholesterol and triglyceride levels compared with the HFD control, supporting the lipid-lowering activity of this dose [60]. However, the effect of ATO 5 mg/kg BW/day on the protection of kidney injury in an obese model has not been extensively investigated. Therefore, ATO 5 mg/kg BW/day was selected as a lower ATO dose which demonstrated lipid-lowering effects to avoid undesired adverse effects in an HFD rat model. This dose was combined with C3G to investigate whether the addition of C3G could provide complementary effects when ATO was administered at half the dose of the ATO monotherapy reference group. Accordingly, ATO 10 mg/kg BW/day was used as the reference treatment (HFA10), whereas ATO 5 mg/kg BW/day was combined with C3G at 100 or 200 mg/kg BW/day to evaluate the metabolic and renal effects of the combination treatment. Body weight and food intake were monitored weekly. Oral glucose tolerance tests (OGTTs) were performed at weeks 16 and 24. At the end of the 24-week experimental period, rats were fasted overnight, anesthetized with isoflurane and euthanized. Blood, visceral fat and kidney samples were collected for further analysis. The experimental protocol is illustrated in Figure 7.

### 4.3. Oral Glucose Tolerance Test (OGTT)

Following an overnight fast, baseline blood glucose was collected from the tail vein at 0 min. Then, rats received an oral glucose load and blood glucose was collected at 30, 60 and 120 min post-administration from the tail vein. Blood samples were centrifuged to obtain plasma and glucose levels were quantified using a colorimetric assay kit (Erba Diagnostics, Miami, FL, USA) at 505 nm. The total area under the curve (AUC) of glucose was calculated using the trapezoidal method.

### 4.4. Plasma Lipid Profile

Blood was collected into ethylenediaminetetraacetic acid (EDTA)-coated tubes for metabolic analysis. Plasma HDL was measured using an automated clinical chemistry analyzer using enzymatic colorimetric methods at the Associated Medical Sciences Clinical Service Center, Chiang Mai University. The levels of plasma triglycerides and total cholesterol were determined using colorimetric assay kits (Erba Diagnostics, Miami, FL, USA) at an absorbance of 505 nm.

### 4.5. Renal Function

Serum creatinine was measured using an automated clinical chemistry analyzer using enzymatic colorimetric methods at the Small Animal Hospital, Chiang Mai University. Estimated glomerular filtration rate (eGFR) was calculated in accordance with the equation eGFR (mL/min) = [urine creatinine (mg/mL) × urine flow rate (mL/min)]/serum creatinine (mg/dL). Urinary protein levels were measured using the colorimetric Bradford protein assay (Bio-Rad Laboratories, Hercules, CA, USA), with bovine serum albumin used as the standard. Absorbance was read at 595 nm.

### 4.6. Renal Lipid Content

Renal cortical tissue was homogenized in isopropanol 1 mL for lipid extraction. The homogenate was centrifuged at 8000 rpm for 15 min at 4 °C, and the supernatant was collected to enable measurement of triglyceride and total cholesterol concentrations using colorimetric assay kits (Erba Diagnostics, Miami, FL, USA). Absorbance was measured at a wavelength of 505 nm.

### 4.7. Renal Lipid Peroxidation

Renal cortical tissue was homogenized in CelLytic™ MT buffer(Merck Millipore, Burlington, MA, USA) supplemented with 1% protease inhibitor and centrifuged at 1600 *g* for 10 min at 4 °C. The supernatant was used to determine MDA levels using a thiobarbituric acid reactive substances (TBARS) assay kit (Cayman Chemical, Ann Arbor, MI, USA), with absorbance measured at 532 nm.

### 4.8. Western Blot Analysis

Renal cortical tissue was homogenized in CelLytic™ MT buffer (Merck Millipore, Burlington, MA, USA) with protease inhibitors and centrifuged at 5000× *g* for 10 min, then 12,000× *g* for 20 min at 4 °C to obtain whole-cell lysates. Mitochondrial lysates were prepared by differential centrifugation at 12,000× *g* for 5 min and 10 min at 4 °C. Both lysates were stored at −80 °C. Protein concentration was determined by Bradford assay (Bio-Rad Laboratories, Hercules, CA, USA). Absorbance measurements were taken at 595 nm using a Bio-Rad iMark™ microplate reader(Bio-Rad Laboratories, Hercules, CA, USA). Equal amounts of protein were separated by sodium dodecyl sulfate–polyacrylamide gel electrophoresis (SDS-PAGE) and transferred to polyvinylidene fluoride (PVDF) membranes. Membranes were blocked with fast-blocking buffer (Visual Protein, Taiwan) for 3 min at room temperature and incubated overnight at 4 °C with primary antibodies diluted in tris-buffered saline (TBS) or phosphate-buffered saline (PBS) containing 0.1% Tween 20. Proteins related to renal lipid metabolism including CD36 (1:750, Affinity Biosciences, Changzhou, Jiangsu, China), PPARα and FAS (1:500, Thermo Fisher Scientific, Waltham, MA, USA) were evaluated. For mitochondrial homeostasis, the protein expression of Sirt-1 (1:500, Cell Signaling Technology, Danvers, MA, USA), PGC-1α (1:500, Merck Millipore, Burlington, MA, USA), Parkin and MFN2 (1:500, Cell Signaling Technology, Danvers, MA, USA) were examined. To assess oxidative stress, the levels of expression of Keap1 (1:1000), HO-1 (1:500), GPX4 (1:1000) (Cell Signaling Technology, Danvers, MA, USA), Nrf2 (1:1000), and NOX4 (1:500) (Santa Cruz Biotechnology, Dallas, TX, USA) were determined. To evaluate kidney injury, the expressions of Kim-1 (1:500, Thermo Fisher Scientific, Waltham, MA, USA) and E-cadherin (1:500, Cell Signaling Technology, Danvers, MA, USA) were measured. Then membranes were incubated with horseradish peroxidase (HRP)-conjugated secondary antibodies for 1 h. Detection was performed via enhanced chemiluminescence (Bio-Rad Laboratories, Hercules, CA, USA), with visualization carried out using the iBright imaging System (Thermo Fisher Scientific, Waltham, MA, USA), and densitometry was quantified using ImageJ software (version 1.53; National Institutes of Health, Bethesda, MD, USA). β-actin (1:10,000) and voltage-dependent anion channel (VDAC) (1:1000) (Cell Signaling Technology, Danvers, MA, USA) were used as a positive control.

### 4.9. Histopathological Assessment

To evaluate kidney and adipose tissue morphology, kidney and visceral fat samples were transversely bisected, fixed in 10% neutral-buffered formalin, and embedded in paraffin. Thin sections were prepared, mounted on glass slides, and stained for microscopic examination. H&E-staining was utilized to assess overall tissue structural and morphological alterations. Kidney structural changes were scored using a modified method adapted from a previously established protocol [61]. PAS staining was performed to quantify glomerulosclerosis, following the method described in a previous study [21]. Tubulointerstitial fibrosis and collagen accumulation were evaluated via Masson’s trichrome staining, using a Masson Goldner staining kit (Merck Millipore, Burlington, MA, USA). Collagen-positive areas within the renal tissue, including glomerular and tubulointerstitial regions, were quantified. Staining within tubular lumens, considered unrelated to tissue collagen deposition, was excluded. All stained slides were examined and analyzed under a Leica DM750 light microscope (Leica Microsystems, Wetzlar, Germany).

### 4.10. Immunohistochemistry

Kidneys were fixed in 10% formalin, embedded in paraffin, and sectioned. After deparaffinization, antigen retrieval was achieved by heating the sections in Tris-EDTA buffer at 93 °C for 20 min. Sections were then incubated with 0.3% Triton X-100 in PBS for 1 h. To block endogenous peroxidase activity and reduce nonspecific binding, the sections were treated with 0.3% hydrogen peroxide and 5% fetal bovine serum, respectively. The slides were incubated overnight at 4 °C with a primary antibody targeting CD36 (1:200; Affinity Biosciences, Changzhou, Jiangsu, China) followed by 1 h of incubation at room temperature with a biotinylated goat anti-rabbit IgG secondary antibody (Merck Millipore, Burlington, MA, USA). Signal detection was performed using horseradish peroxidase (HRP) reagent (Merck Millipore, Burlington, MA, USA) and diaminobenzidine tetrahydrochloride (DAB; HiMedia Laboratories, Mumbi, India). Finally, the sections were counterstained with hematoxylin (HiMedia Laboratories, Mumbi, India). CD36-positive staining was observed under a Leica DM750 light microscope (Leica Microsystems, Wetzlar, Germany) in a blinded manner and quantified with ImageJ software (version 1.53; National Institutes of Health, Bethesda, MD, USA).

### 4.11. Statistical Analysis

Data are expressed as mean ± standard deviation (SD). Statistical analyses were carried out using GraphPad Prism version 10 (Dotmatics, Boston, MA, USA). Data normality was assessed using the Kolmogorov–Smirnov test, and no significant deviation from normality was detected (*p* > 0.05). Comparisons between two groups were performed using an independent *t*-test, whereas multiple-group comparisons were analyzed using one-way analysis of variance (ANOVA), followed by Fisher’s least significant difference (LSD) post hoc test. Statistical significance was defined *p* < 0.05.

## 5. Conclusions

Treatment with C3G in combination with ATO (5 mg/kg BW/day) was associated with attenuation of obesity-induced kidney injury, with favorable effects across metabolic, renal, and molecular outcomes. In the initial dose comparison, both C3G doses (100 and 200 mg/kg BW/day) combined with ATO 5 mg/kg BW/day were evaluated. HFC100A5 was selected as the lower C3G dose for subsequent molecular analyses. At the C3G 100 mg/kg BW/day dose, combination treatment with ATO 5 mg/kg BW/day was associated with reduced renal lipid accumulation and favorable changes in proteins related to lipid metabolism, mitochondrial homeostasis, and oxidative stress. Specifically, HFC100A5 increased PPARα, Sirt-1, and MFN2 expression, with upward trends in PGC-1α and Parkin, suggesting favorable changes in lipid metabolism and mitochondrial homeostasis. HFC100A5 also significantly reduced renal NOX4 expression and MDA levels and increased Keap1, Nrf2, and HO-1 expression, with an upward trend in GPX4, indicating favorable changes in oxidative stress and antioxidant defense. Notably, compared with ATO 10 mg/kg BW/day alone, HFC100A5 showed significantly lower renal NOX4 and MDA levels and significantly higher MFN2 expression, whereas ATO 10 mg/kg BW/day alone did not significantly reduce NOX4 expression. These findings highlight favorable changes in selected markers of oxidative stress and mitochondrial homeostasis with the combination treatment compared with the higher ATO dose alone. These molecular changes were accompanied by preservation of renal structure and improvement in selected indicators of renal function. Across several measured metabolic and renal outcomes, the effects of HFC100A5 were broadly comparable to those observed with HFA10. Overall, the findings suggest that C3G combined with ATO 5 mg/kg BW/day was associated with coordinated improvements in renal lipid metabolism, mitochondrial homeostasis, and oxidative stress, which may collectively contribute to attenuation of obesity-associated kidney injury. The data obtained from this study support further investigation of C3G combined with ATO as a potential adjunctive strategy for obesity-associated kidney injury.

## Data Availability

The datasets analyzed in this study are not publicly available but are available from the corresponding author upon reasonable request. The data can be provided for legitimate research purposes, subject to appropriate review and approval.

## Abbreviations

The following abbreviations are used in this manuscript:

C3G	Cyanidin-3-*O*-glucoside
ATO	Atorvastatin
